# 2247. Improvements in Antibiotic Stewardship for Patients Who Use Injection Drugs: Subgroup Analysis of Patients with Bacteremia

**DOI:** 10.1093/ofid/ofad500.1869

**Published:** 2023-11-27

**Authors:** Olivia Duffield, Kaya Patel, Sara K Schultz, Stephanie Spivack

**Affiliations:** Temple University School of Medicine, Philadelphia, Pennsylvania; Temple University Hospital, Philadelphia, Pennsylvania; Temple University Hospital, Philadelphia, Pennsylvania; Temple University Health System, Philadelphia, Pennsylvania

## Abstract

**Background:**

Infectious complications are common in patients who inject drugs (PWID). In this population, infections include acute bacterial skin and skin structure, bacteremia, endocarditis, and osteomyelitis. Despite low rates of gram-negative (GN) infections, coverage for these organisms is often initiated on presentation. Herein, we describe the microbiological and clinical characteristics of PWID admitted to our large academic medical center in Philadelphia with a focus on GN bacteremia.

**Methods:**

We queried Epic for admissions with patients that had documented opioid use disorder with injection behavior and positive blood cultures from September of 2021 to March of 2022. We conducted a retrospective chart review of these encounters and collected data regarding their hospital course and treatment.

**Results:**

During the six-month study period, we reviewed 159 encounters of PWID with bacteremia on admission. Of these encounters, 156 (98%) patients had gram-positive (GP) organisms in their blood culture, 12 (7.5%) patients had GN organisms. Of the GP organisms, MRSA (65, 41%), MSSA (27,17%) and Group A streptococcus (53, 33%) were the dominant organisms. Of the patient encounters with GN bacteremia, 4 (33%) encounters were found non-attributable to injection behavior (2 in the setting of gastrointestinal infection, and 2 attributed to pneumonia without presence of septic pulmonary emboli). Three (20%) were found to be secondary to necrotizing soft tissue infections. Five (41%) patients with GN bacteremia left the hospital prior to identification of source. In addition to the 159 encounters for bacteremia, there were an additional 78 (33%) encounters with positive blood cultures later determined to be a contaminant. 61 of 78 encounters (78.2%) had timely discontinuation of antibiotics upon speciation of contaminant blood culture.

Figure 1

**Figure:**
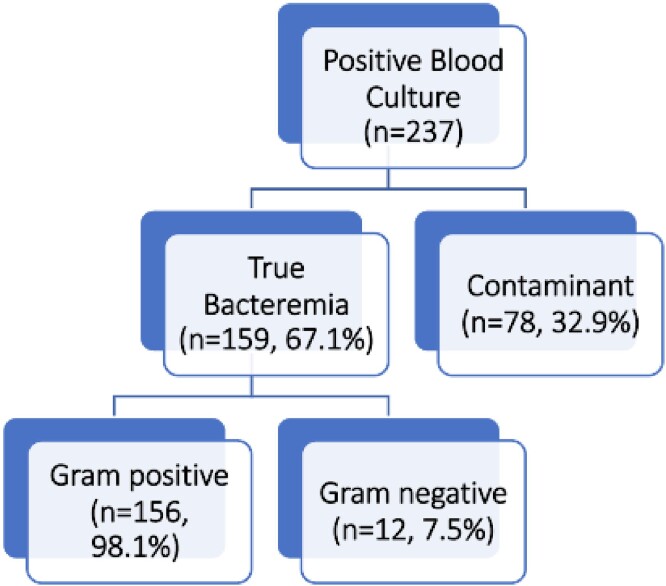

Characteristics of Positive Blood Cultures

**Conclusion:**

Although PWID frequently present with infectious symptoms with bacteremia, GN bacteremia occurs in a minority of infections, challenging the notion that patients require empiric GN coverage. Furthermore, a notable percentage of GN bacteremia is secondary to a source non-attributable to injection behavior. We are continuing to explore why this population may have a high rate of contaminant blood cultures.

**Disclosures:**

**Sara K. Schultz, MD FACP FIDSA**, AbbVie: Advisor/Consultant

